# Early and parallel processing of pragmatic and semantic information in speech acts: neurophysiological evidence

**DOI:** 10.3389/fnhum.2013.00086

**Published:** 2013-03-28

**Authors:** Natalia Egorova, Yury Shtyrov, Friedemann Pulvermüller

**Affiliations:** ^1^Medical Research Council, Cognition and Brain Sciences UnitCambridge, UK; ^2^Brain Language Laboratory, Department of Philosophy and Humanities, Freie Universität BerlinBerlin, Germany

**Keywords:** speech act, pragmatics, communicative action, social interaction, electroencephalography (EEG), L1 norm source reconstruction, fronto-central cortex, temporo-parietal cortex

## Abstract

Although language is a tool for communication, most research in the neuroscience of language has focused on studying words and sentences, while little is known about the brain mechanisms of speech acts, or communicative functions, for which words and sentences are used as tools. Here the neural processing of two types of speech acts, Naming and Requesting, was addressed using the time-resolved event-related potential (ERP) technique. The brain responses for Naming and Request diverged as early as ~120 ms after the onset of the critical words, at the same time as, or even before, the earliest brain manifestations of semantic word properties could be detected. Request-evoked potentials were generally larger in amplitude than those for Naming. The use of identical words in closely matched settings for both speech acts rules out explanation of the difference in terms of phonological, lexical, semantic properties, or word expectancy. The cortical sources underlying the ERP enhancement for Requests were found in the fronto-central cortex, consistent with the activation of action knowledge, as well as in the right temporo-parietal junction (TPJ), possibly reflecting additional implications of speech acts for social interaction and theory of mind. These results provide the first evidence for surprisingly early access to pragmatic and social interactive knowledge, which possibly occurs in parallel with other types of linguistic processing, and thus supports the near-simultaneous access to different subtypes of psycholinguistic information.

## Introduction

Research in the neuroscience of language has so far mainly focused on the brain basis of words and utterances. However, the main function of language is to allow communication and there is not a one-to-one relationship between utterances and their function in communicative interactions. The same words or utterances can be tools for different communicative functions. For example, the sentence “Here is an apple” can be used to *teach* somebody the meaning of the word, to *direct* somebody's *attention* to a particular object, or to *offer* it upon request. Clearly, it is the situation and context, that is, pragmatic information that determines these communicative functions and the way the utterance is typically understood. It remains largely unknown how this communicative function is represented in the human brain and when its processing takes place in language comprehension. This study aims to take a first step in exploring the brain basis of communicative functions, or the so-called speech acts, for which linguistic utterances serve. Importantly, it attempts to establish the neural time course of comprehension of communicative functions, and situates pragmatic processing in relation to other types of psycholinguistic information access. To this end, an experiment was conducted, comparing the brain responses in subjects watching video clips with Naming or Request interactions, in which the same single word utterances were used to perform these speech acts. The neurophysiological responses are used to draw conclusions about the time course and cortical loci of brain processes supporting speech act understanding.

### Speech act processing

**Figure 1:**
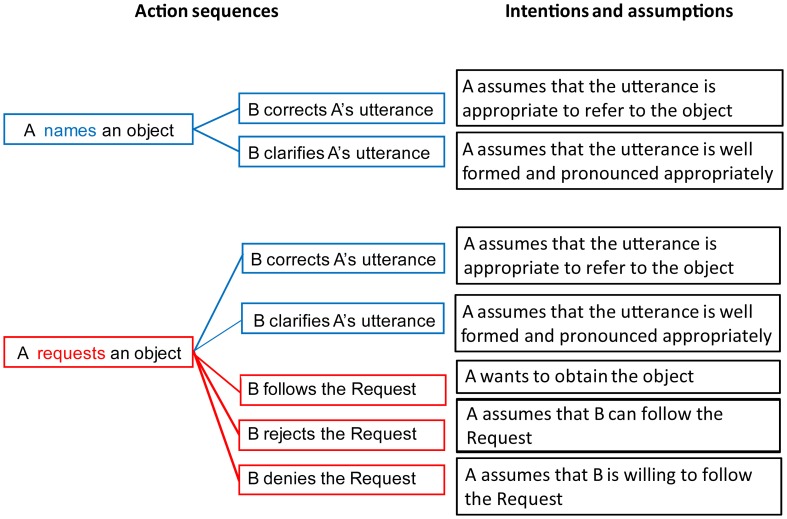
**Comparison of pragmatic properties of the speech acts of Naming (Top) and Requesting (Bottom).** Action sequence schemes **(Left)** show typical actions following the speech acts, which are closely linked to the intentions and assumptions **(Right)** characterizing the speech acts.

A speech act (Austin, 1962; Searle, 1969; Van Dijk, 1977; Fritz and Hundsnurscher, 1994; Horn and Ward, 2006; Ehlich, 2010) can be characterized by specifying (1) the *linguistic utterance* used to perform it; (2) the *physical setting* in which the communicative actors find themselves; (3) the *intentions and assumptions* the actors commit themselves to; and (4) the *action sequence structures* in which the speech act is typically embedded (i.e., actions occurring with the speech act, including those preceding and following it). As there are many different speech acts, (for a systematic classification see Bach and Harnish, 1979; Searle, 1979), we here focus on two prototypical examples, the speech acts of Naming and Requesting (Figure 1).

In a communicative situation involving two people (a Speaker and a Partner), the use of the word “Water” by the Speaker while pointing at a glass of water on a table can be understood by the Partner as NAMING the liquid in the glass. However, the same word utterance in the same context can alternatively be understood as a REQUEST to give the glass of water to the Speaker. In this situation, the linguistic utterance (1) and the physical setting (2) will be identical for the two speech acts, because the same single word is used, and the same physical object is available. However, other aspects of the context, including the assumptions and intentions of both communicating partners, as well as the structure of the expected action sequences, differ between the two speech acts.

In the situation of Naming, the Speaker produces a linguistic utterance appropriate for referring to the object in question. The assumptions associated with the utterance include (but are not limited to) the Speaker knowing the name of the object and willingness to communicate it to the Partner, pronouncing it correctly, referring to an appropriate object, and so on. Accordingly, potential Partner's actions can possibly involve Clarifying the name, Correcting, or Confirming it verbally (i.e., suggesting a different name or agreeing with the Speaker) or non-verbally (a negative shrug or a positive nod).

On the other hand, in the situation of Requesting, the Speaker typically indicates an object that she or he wants to obtain from the Partner. The assumptions behind the speech act of Requesting include those typical of Naming, such as the correct use of the word and clear articulation from the Speaker. In addition, there are assumptions specific to a Request, for instance the Speaker's intention to get the object. Crucially, the assumptions characteristic of a Request include additional ones about the Partner, namely, the Partner's ability to perform the required action (here—to hand over the requested object), as well as his/her willingness to do so. With a growing number of assumptions associated with a Request, there is also a wider range of actions potentially following the speech act of Request. Similarly to Naming, the Partner can respond to a Request by asking for a clarification of the linguistic utterance if the latter is not well-formed, by correcting the use of the word, or by complaining about the absence of the requested object. Additionally to the responses typically following Naming, the Partner can choose to perform the required action, i.e., pass the object over to the Speaker; or refuse to perform the required action, if the assumption that they are able or allowed to do so is not satisfied; or they can choose to deny it, in case they are fully capable of performing the action but are not willing to do so. The assumptions and intentions characterizing Naming and Request actions are illustrated, along with the associated structures of action sequences, in Figure 1. Note that both speech acts are social and can be followed by actions, however, compared with Naming, Requests involve more action (e.g., that an object needs to be manipulated) and social interaction knowledge (e.g., recognizing the Speaker's desire to obtain the object).

These premises allow generating theoretical predictions also for the neurophysiology of speech act processing. First, Requests may activate brain areas for processing of action and social-interaction knowledge more than Naming. These areas are in the fronto-central sensorimotor system (Pulvermüller and Fadiga, 2010) and in the temporo-parietal junction (TPJ) (Van Overwalle and Baetens, 2009), respectively. Stronger activation during Naming compared with Request might, in turn, be expected in areas important for linking linguistic representations with visual objects representations. Such semantic areas that might be of special importance for processing the referential link between words and objects are in the middle temporal cortex and other parts of the temporal lobe (Damasio et al., 1996; Pulvermüller and Fadiga, 2010). Finally, left perisylvian and visual word form area activations may be expected during both Naming and Request, as written language stimuli are used in this study. These predictions were experimentally tested here by contrasting Naming and Request action performed with the same words, embedded in similar physical settings but with different communicative functions.

### Timecourse of speech act processing

This study also addresses the issue of the time course of psycholinguistic information access. Based on the relative time course of semantic and pragmatic information access, it is possible to compare serial or cascaded models of processing (postulating sequential access to phonological, lexical, semantic, syntactic, and pragmatic information each taking about 100 ms to complete; see e.g., Garrett, 1980; Friederici, 2002; Pickering and Garrod, 2004) and near-simultaneous processing models (assuming parallelism in the processing of different types of linguistic information, taking place within 200 ms from the stimulus onset, and followed by the second stage of semantic and syntactic reanalysis and second-order cognitive processing between 200 and 600 ms Marslen-Wilson, 1975; Marslen-Wilson and Tyler, 1980; Pulvermüller, 1996; Pulvermüller et al., 2009; Shtyrov, 2010). To identify relative time course of single-word semantic and pragmatic processing, critical words (concrete nouns) of two semantic categories (“Hand-related” and “Non-Hand-related”) were used as stimuli. The *Hand* type comprised the words denoting functionally manipulable objects (e.g., “spoon,” manual action is necessary to use the object) and *Non-Hand* type included words denoting objects that do not require manual actions (e.g., “plant,” although manual action is possible, it is not necessary), as used in previous work (Rueschemeyer et al., 2010); thus, semantic and pragmatic factors were orthogonally manipulated.

Serial/cascaded models predict that semantic processes start before pragmatic processes, and thus a sequence/cascade of physiological effects, with no (or only minimal) interactions between the processing stages, is expected. In this experiment, a serial-onset model would predict differences between semantic categories identified first, likely around 200 ms, and the differences between the speech act types first present later, after 400 ms. According to the model of near-simultaneous processing of psycholinguistic information (Pulvermüller et al., 2009), the process of integrating speech act information should take place in parallel with lexical-semantic information access. This view predicts that both the semantic and the pragmatic manipulations will be reflected in neural activation before or around 200 ms, likely with an interaction between semantic and pragmatic levels. The differences will persist at a later time window of about 400 ms, at least if second-level post-comprehension processes are encouraged by the experimental paradigm.

## Materials and methods

### Subjects

Twenty healthy right-handed volunteers took part in the study. Two participants were excluded from further analysis: one because of left-handed first-order relatives, and the other based on poor performance on the behavioral task (low accuracy and fast reaction times). Additionally, the behavioral data from one participant were not collected due to software failure. Thus, the final sample consisted of 18 participants (12 females, mean age = 26, range 18–40, *SD* = 6.8), with 17 of them contributing to the reported behavioral measure. The participants were all monolingual native speakers of British English. Informed consent was obtained from the participants, and they were paid for taking part in the experiment. All were right-handed, according to the Edinburgh Handedness Inventory (Oldfield, 1971), mean laterality coefficient of 80.5 (range 53–100, *SD* = 16.8), without left-handed first-order relatives. A measure of general intelligence was obtained, using the Cattell Culture Fair Test, Scale 2 Form A [Institute for Personality and Ability Testing, 1973 (Cattell, 1971)]; the group's mean Cattell score was 36 (range 27–42, *SD* = 4.5). Ethical approval was obtained from the Cambridge Local Research Ethics Committee.

### Stimuli

**Figure 2:**
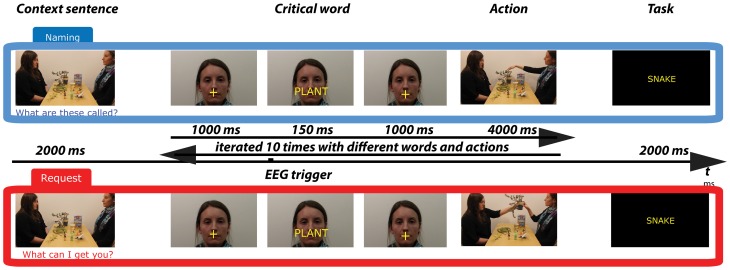
**Schematic illustration of trials in the Naming (Top) and Request (Bottom) conditions.** A trial sequence starts with a display of objects and communicating actors. A context sentence (e.g., “What can I get you?”) is uttered by the Partner. Following this, a series of 10 loops are presented, in which the Speaker's face appears together with the critical word-utterance (naming vs. requesting an object), followed by an action (handing over or pointing at the object), involving different words and objects. The trial sequence finishes with a task to press a button (yes-no) if the test word shown on the screen has appeared in the trial.

The stimuli consisted of two sets of 16 experimental videos displaying one trial sequence (see Figure 2). Each video featured two persons (a “Partner” and a “Speaker”) sitting by a table in front of each other and 12 objects lying on the table. Each video started with the *context sentence* pronounced by the Partner determining the speech act for which the *critical word* pronounced by the Speaker was used. Then, 10 trials followed, each including the utterance of a critical word and a non-verbal action following it. The 10 words were names of 10 out of the 12 objects on the table. As determined by the context sentence, the utterance of the critical word was either a Naming or a Request speech act. The average duration of videos was 93 s (range 76–114, *SD* = 12). Three male and three female speakers were used to record the videos. Two of them were “Partners,” and four were “Speakers” (gender-balanced). The positions of the Partner and Speaker in relation to one another (left-right) were counterbalanced. The videos were processed using Adobe Premier Pro software (Adobe Systems Incorporated, San Jose, California, USA). The two sets of experimental videos containing the same critical words and objects, but differing in the speech act type, were presented in two lists counterbalanced between groups. The details on the content of the videos and the stimuli selection are described below.

Three pairs of matched sentences (three providing the context for the speech act of Naming, e.g., “Which of these things can you name?” and three for the speech act of Request, e.g., “Which of these things would you like?”) were used. These six sentences were pseudo-randomly used in 16 trials. The sentences in each pair were matched on the number of words and complexity. They represented different syntactic types (interrogative, imperative).

One hundred and sixty monosyllabic words (from noun categories: food, tools, animals, clothes, everyday objects) and corresponding objects were selected from two semantic types, Hand-related and Non-Hand-related. A rating study based on 7-point Lickert scale was run with 10 native English speakers as participants to empirically assess semantic properties of the stimulus words: imageability, concreteness, arousal, valence, potency, association with action, manipulability, and visual movement (i.e., the movement that can be perceived visually, such as that of a flying bird). The ratings confirmed the a priori classification of stimulus words as either strongly Hand-related or Non-Hand-related and the absence of differences along other semantic properties. The two groups were also matched on the number of letters, word form and lemma frequency (linear and logarithmic), the number of orthographic neighbors (words that can be derived from a given word by exchanging one letter), and orthographic bigram and trigram letter frequency using CELEX (Baayen et al., 1993). All words were either lexically unambiguous nouns or, in case of lexical ambiguity, predominantly used as nouns (for details, see Table 1).

**Table 1:** **Psycholinguistic and semantic properties of Hand-related and Non-Hand-related word stimuli**.

**A.**	**NL**	**WF**	**Log WF**	**LF**	**Log LF**	**OBF**	**OTF**	**ONS**
Mean—Hand (SE)	4.22 (0.09)	25.91 (4.27)	1.17 (0.05)	55.43 (8.75)	1.51 (0.05)	36255.38 (1982.75)	3486.57 (266.15)	8.55 (0.66)
Mean—Non-Hand (SE)	4.18 (0.09)	25.93 (4.45)	1.14 (0.05)	60.84 (8.90)	1.51 (0.05)	35968.12 (1984.95)	3721.68 (281.42)	8.60 (0.68)
*p*-value (*t*-test, 2-tailed)	0.71 ns	1.00 ns	0.77 ns	0.67 ns	0.96 ns	0.92 ns	0.54 ns	0.96 ns
**B.**	**NM**	**Noun**	**Verb**	**Other**	**WF (as Noun)**	**WF (as Verb)**	**LF (as Noun)**	**LF (as Verb)**
Mean—Hand (SE)	0.28 (0.05)	1 (0)	0.71 (0.05)	0.04 (0.02)	24.39 (5.03)	0.98 (0.40)	50.52 (13.03)	25.88 (11.7)
Mean—Non-Hand (SE)	0.35 (0.07)	1 (0)	0.68 (0.05)	0.05 (0.03)	26.40 (4.68)	1.05 (0.38)	56.56 (10.36)	6 (9.02)
*p*-value (*t*-test, 2-tailed)	0.51 ns	1.00 ns	0.69 ns	0.77 ns	0.77 ns	0.89 ns	0.71 ns	0.99 ns
**C.**	**Action**	**Hand**	**Movement**	**Imageability**	**Concreteness**	**Arousal**	**Valence**	**Potency**
Mean—Hand (SE)	4.45 (0.10)	4.55 (0.13)	4.44 (0.12)	6.47 (0.05)	6.64 (0.04)	2.75 (0.11)	4.27 (0.08)	3.92 (0.09)
Mean—Non-Hand (SE)	3.33 (0.12)	2.86 (0.15)	3.72 (0.11)	6.42 (0.05)	6.68 (0.05)	2.82 (0.11)	4.38 (0.07)	3.93 (0.09)
*p*-value (*t*-test, 2-tailed)	<0.001 ^***^	<0.001 ^***^	<0.001 ^***^	0.53 ns	0.54 ns	0.68 ns	0.33 ns	0.97 ns

Mean values, Standard Error of Mean and significance p-values are shown. ***(A)*** Psycholinguistic properties. NL, Number of letters; WF, Word form frequency; Log WF, Logarithm to base 10 of WF; LF, Lemma frequency; Log LF, Logarithm to base 10 of LF; OBF, Orthographic bi-gram frequency; OTF, Orthographic tri-gram frequency; ONS, Orthographic neighborhood size. ***(B)*** Use as members of lexical classes. NM, Number of meanings; Noun, Proportion of use as a noun; Verb, Proportion of use as a verb; Other, Proportion of use as other parts of speech; WF (as Noun), Word form frequency as a Noun; WF (as Verb), Word form frequency as a Verb; LF (as Noun), Lemma frequency as a Noun; LF (as Verb), Lemma frequency as a Verb. ***(C)*** Semantic ratings (7-point Lickert Scale). Action, Action-relatedness; Hand, Hand-relatedness; Movement, Visual movement-relatedness; Imageability; Concreteness; Arousal; Valence; Potency.

In addition to the 160 objects denoted by the matched words, 32 objects were added as fillers used for the experimental task. This was done to exclude the possibility that the participants could infer with absolute certainty, which objects would be named/requested by the end of each multi-item trial. All the objects were divided into 16 sets of 12 objects (10 relevant critical words and 2 fillers), so that in each set there were objects of both semantic types as well as fillers, and that the objects varied in size within and across sets.

After the utterance of the critical word in 80% of the cases, the typical action followed, i.e., handing over of the requested object in the Request condition and pointing to the named object in the Naming condition. In 20% of the cases, either a verbal clarification (“What did you say?” or “Could you repeat it please?” uttered by the Partner) or a gestural refusal (negative headshaking/shrug by the Partner) occurred. These additional actions were introduced to closely approximate the context in which Naming and Request typically occur, as well as to ensure that the particular speech act can only be determined with certainty at the critical word and not before it.

### Procedure

The stimuli were visually presented using E-prime 2.0 (Psychology Software Tools, Pittsburgh, PA). The participants were asked to attend to the experimental stimulation using visually presented instructions, and, to ensure their attention on the stimulation, were warned that they would be tested on its content. The participants were told that they would see videos of two people interacting, and that one of them would ask the other to name the objects on the table, or to ask for these objects, and that the speakers can only answer “in one word.” Single word use allowed avoiding the use of articles, which could differ between stimuli and could introduce additional variability in the event-related potential (ERP) signal.

After the instructions, 16 trials, each followed by a task, were presented as described in Figure 2. The video with a spoken context sentence was shown, but the critical words were presented visually against the background of the Speaker's face (on the lips) for 150 ms followed by a video of an action of pointing to or handing over the object named or requested using the critical word. Each context sentence was followed by 10 word-action pairs.

Finally, to test the participants' attention on the visual input, they were asked to press the button “yes” or “no” (left/right, counterbalanced between subjects) to indicate whether the test word presented after the video in the middle of the screen for 2000 ms had been mentioned in the video. Accuracy and reaction times were recorded.

### EEG recording

The electroencephalogram (EEG) was measured in an electrically and acoustically shielded EEG chamber at the MRC Cognition and Brain Sciences Unit in Cambridge, UK. Data were recorded from 128 Ag/AgCl electrodes mounted on the EEG cap (actiCAP, Brain Products, Gilching, Germany). The electrodes were arranged according to the extended 10/20 system. Data were sampled at 500 Hz with a band-pass filter 0.1–100 Hz. FCz was used as recording reference for the EEG channels. The EOG was recorded bipolarly through electrodes placed above and below the left eye (vertical) and at the outer canthi (horizontal).

### Data analysis

The acquired EEG data were processed offline using Brain Vision Analyzer 1.05 (Brain Products) software. They were bandpass-filtered at 1–40 Hz with a notch filter of 50 Hz, segmented into epochs starting 100 ms before the onset of the word to 800 ms thereafter, with the baseline corrected using −100 to 0 ms interval before the stimulus onset. Epochs with signal exceeding −75 and 75 μV were discarded and data were re-referenced to the average mastoids. The time windows for the analysis were defined using the top quarter of amplitude of the peaks observed in the global field power (GFP) waveform calculated on the selection of electrodes (see below) and showing activation collapsed over all four conditions averaged across all participants. Following this, three types of analysis were used.

Firstly, the statistical analysis was performed on the GFP mean amplitudes to determine any global effects of the pragmatic and semantic conditions. An ANOVA with Pragmatics (two levels: Naming and Request) × Semantics (two levels: Non-Hand category and Hand category) was performed for each time window separately.

**Figure 3:**
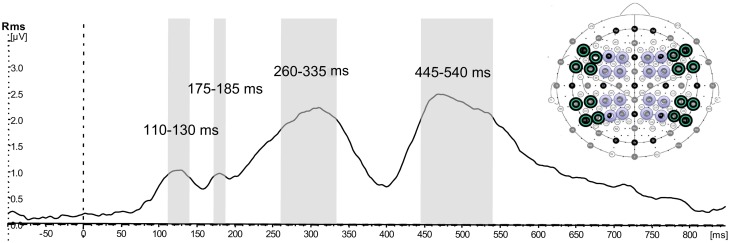
**Selection of time windows and electrodes for the topographical analysis.** Global field power (GFP) calculated on the selection of 32 electrodes showing activation collapsed over all four conditions averaged across all participants, and illustration of time windows and electrodes included in further topographical signal-space analysis: green—peripheral, purple—central.

The second step of the statistical analysis was performed using response amplitudes in a selection of electrodes in order to evaluate the topography of the activations. For the statistical analysis in signal space a selection of 32 electrodes (see Figure 3), where activity was strongest, was made. The electrodes were divided into anterior (F7, F5, F3, F1, F2, F4, F6, F8; FT7, FC5, FC3, FC1, FC2, FC4, FC6, FT8) and posterior (TP7, CP5, CP3, CP1, CP2, CP4, CP6, TP8; P7, P5, P3, P1, P2, P4, P6, P8). These were further divided into left-hemispheric (odd electrode numbers) and right-hemispheric (even electrode numbers), and within each hemisphere they were divided into more peripheral (lines 7,5,6,8) vs. more central (lines 3,1,2,4). This electrode selection allowed contrasting a number of topographical factors—anteriority (anterior-posterior), laterality (left-right), as well as centrality (central-peripheral). Separate ANOVAs with the factors Pragmatics (2) × Semantics (2) × Anteriority (2) × Laterality (2) × Centrality (2) were performed for each of the time windows. A Huynh-Feldt correction was applied whenever there was more than one degree of freedom in the numerator. For all interactions a separate analysis investigating the contribution of the Semantic and Pragmatic factors at all the levels of the interaction was performed. Significant results of the pairwise comparisons for the pragmatic and semantic conditions, mean values and standard errors are reported.

Additional statistical tests for possible effects in N400 and P600 time ranges often implicated in language ERP studies (350–450 ms and 550–650 ms, respectively) were also performed using procedures identical to the above.

Finally, in order to localize cortical sources of the activations underlying differences between the main conditions (see “Results”), L1 Norm current estimation on ERP difference between the Request and Naming conditions, as well as the Hand and Non-Hand conditions, was performed (using CURRY 6.1 software, Compumedics Neuroscan, Hamburg, Germany). This distributed source analysis method produces only a few active sources per latency and is very sensitive to signal-to-noise ratio (SNR). The solutions were calculated for grand-averaged responses, as this has improved SNR compared to individual source solutions (especially important for EEG source localization, which is more prone to error), and were focused on those latencies where significant effects were found in the statistical analysis above. A three-layer boundary element model (BEM) with triangularized gray matter surface of a standardized brain (Montreal Neurological Institute) was used for computing the source reconstruction solutions. The solutions were restricted to cortex surface. Noise covariance matrix was applied and noise user defined interval was set from −100 to 0 ms. For each of the sources, the putative regions they correspond to and, where available, the Brodmann areas, are reported.

In addition to the ERP analysis triggered by and following the critical words, a separate analysis of the neurophysiological response to the fixation cross preceding the critical word was performed. This was done to check whether there were any neurophysiological differences between Naming and Requests contexts that were independent of the linguistic processes brought about by the critical, speech act carrying linguistic structure. Please recall that, within the time interval preceding each critical word, a fixation cross was presented against the Speaker's face (similarly to the critical words) for the duration of 1000 ms. The analysed epochs starting 100 ms before the onset of the cross to 1000 ms thereafter were processed in the same way as the critical words and analysed similarly to the GFP analysis of the critical words.

## Results

### Behavioral results

Accuracy in the behavioral task was high (Naming: mean 85.3%, *SE* = 3.5; Request: 89.8%, *SE* = 2.6) and did not differ significantly between the pragmatic conditions [*t*_(16)_ = 1.165, *p* > 0.05, 2-tailed]. There were also no significant differences in reaction times (Naming: 1561 ms, *SE* = 131; Request = 1504 ms, *SE* = 131 ms) between the two conditions [*t*_(16)_ = 1.174, *p* > 0.05, 2-tailed].

### EEG results

#### GFP analysis

The GFP waveform showing activation collapsed over all four conditions across all the selected electrodes revealed peaks at 128, 180, 310, and 468 ms (see Figure 3). Time windows were defined around these peaks approximating the top quarter in amplitude of each peak, resulting in the intervals 110–130 ms, 175–185 ms, 260–335 ms, 445–540 ms.

**Figure 4:**
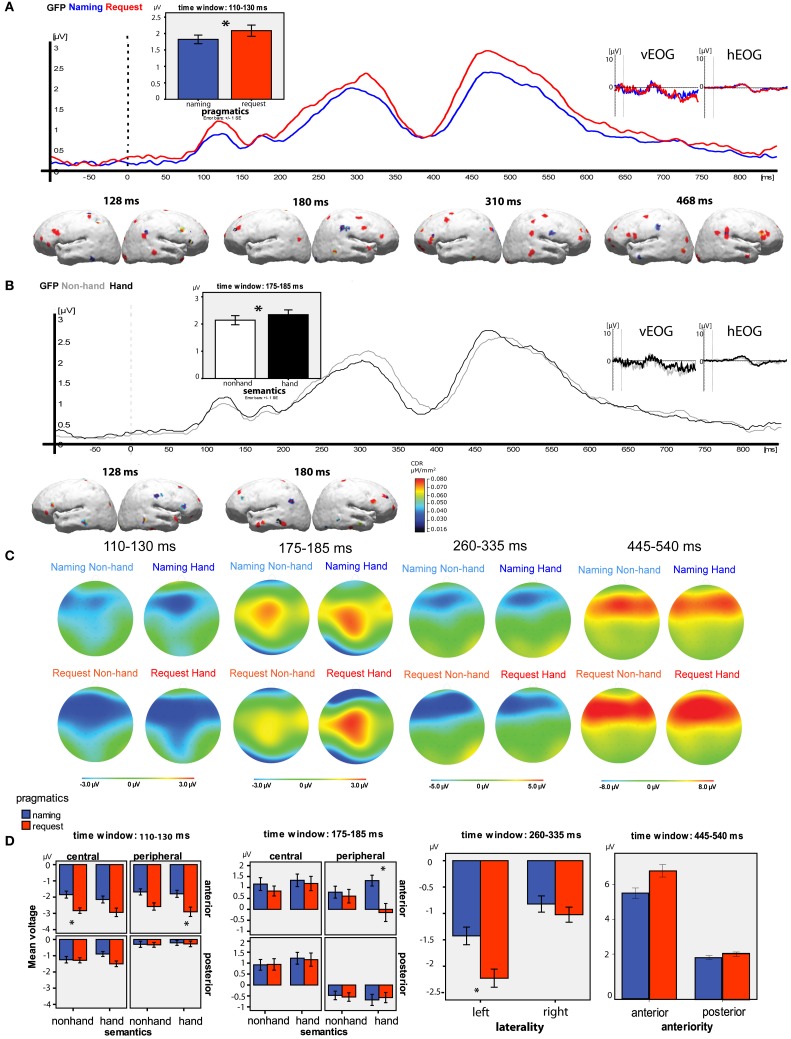
**Summary of main results. (A)** Pragmatic conditions grand average GFP time-locked to the noun onset (blue—naming, red—request); vertical and horizontal EOG grand average curves (no significant differences between the conditions in the analysed time windows); bar graphs illustrating the significant results of the GFP analysis for the pragmatic condition; the results of L1-norm source reconstruction (Request > Naming). **(B)** Semantic conditions grand average GFP time-locked to the noun onset (gray—Non-Hand, black—hand); vertical and horizontal EOG grand average curves (no significant differences between the conditions in the analysed time windows); bar graphs illustrating the significant results of the GFP analysis for the semantic condition; the results of L1-norm source reconstruction (Hand>Non-Hand).**(C)** Topographical plots for each time window and condition. **(D)** Bar graphs illustrating the significant results of the topographical analysis for each time window, the error bars represent Standard Error of the Mean, the asterisks indicate significant differences between the conditions in pairwise comparisons, at *p* < 0.05.

The GFP analysis of Pragmatics × Semantics effects for separate time windows showed the following (see Figures 4A,B). In the time window 110–130 ms, a significant main effect of Pragmatics [Request > Naming, *F*_(1, 17)_ = 5.382, *p* = 0.03], and a near-significant effect of Semantics [Hand > Non-Hand, *F*_(1, 17)_ = 4.265, *p* = 0.054], emerged. In the second time window of 175–185 ms, there was a significant effect of Semantics [Hand > Non-Hand, *F*_(1, 17)_ = 4.400, *p* = 0.05]. The analysis of the third time window of 260–335 ms did not show any significant results; and in the fourth window of 445–540 ms there was only a near-significant effect of Pragmatics, *F*_(1, 17)_ = 0.081, with more positivity observed for Request than for Naming. Additional time windows of the GFP were tested but the statistical analysis did not confirm any significant differences outside the four peaks, including the typical N400 and P600 time ranges.

Analysis of brain activity preceding the critical word (at the presentation of a fixation cross) revealed a similar GFP curve as seen to critical words, with peaks at 80–100 ms; 140–160 ms; 210–250 ms, corresponding to typical visual ERP latencies of N1, P1, N2 components. A repeated measures ANOVA (Semantics × Pragmatics) was performed for each of the three time windows, as for the word-elicited responses. No significant interactions or main effects of either semantic or pragmatic conditions were found in response to the simple cross.

#### Topographical analysis

The results of the ANOVAs performed for the four time windows individually are reported below. The topographical maps of the activations for each of the time windows for each condition are shown in Figure 4C and the main statistical findings per time window are illustrated in Figure 4D.

***Time window 1 (110–130 ms).*** A four-way interaction of Pragmatics, Semantics, Anteriority, and Centrality was observed [*F*_(1, 17)_ = 6.725, *p* = 0.02]. Pairwise comparisons confirmed that the basis of this interaction was the difference between the pragmatic conditions at the anterior central electrodes in the Non-Hand category (Naming −1.848 ± SE 0.517 μV; Request −2.147 ± 0.493 μV; *p* = 0.02) and at the anterior peripheral electrodes in the Hand category (Naming −2.580 ± 0.364 μV; Request −2.906 ± 0.558 μV; *p* = 0.04).

***Time window 2 (175–185 ms).*** Similarly to the first time window, there was a four-way interaction of Pragmatics, Semantics, Anteriority, and Centrality [*F*_(1, 17)_ = 14.935, *p* = 0.001]. Pairwise comparisons showed that there were significant differences between the pragmatic conditions (Naming 1.313 ± 0.551 μV; Request −0.145 ± 0.860 μV; *p* = 0.05) at the anterior peripheral electrodes in the Hand semantic category. This is the only time window and electrode selection where Naming elicited larger ERP amplitudes than Request.

***Time window 3 (260–335 ms).*** A significant interaction of the factors Pragmatics and Laterality was observed [*F*_(1, 17)_ = 6.414, *p* = 0.02], with a greater negativity in response to Request compared to Naming in the left hemisphere (Naming −1.426 ± 0.552 μV; Request −2.227 ± 0.515 μV; *p* = 0.04) but not in the right.

***Time window 4 (445–540 ms).*** A near-significant interaction of the factors Pragmatics and Anteriority was observed [*F*_(1, 17)_ = 4.202, *p* = 0.056]. The pairwise comparisons suggested that it was due to a trend toward higher positive amplitude in the Request condition than in the Naming condition at the anterior sites (Naming 6.117 ± 1.524 μV; Request 7.409 ± 1.666 μV; *p* = 0.07) but not at the posterior ones. Further pairwise comparisons showed that there were significant differences between the pragmatic conditions (higher amplitude for Request) at the anterior left sites (Naming 6.005 ± 1.500 μV; Request 7.612 ± 1.625 μV; *p* = 0.05), and they were most pronounced at the anterior left central sites (Naming 6.107 ± 1.545 μV; Request 8.104 ± 1.770 μV; *p* = 0.04).

### Source reconstruction

Reconstruction of the sources of activation was performed separately for pragmatic and semantic contrasts (see Figures 4A,B, respectively).

For the pragmatic conditions, the difference between Request and Naming was calculated and then used for the reconstruction of cortical sources contributing to the higher Request activation. The sources for the opposite contrast were not calculated, as in the GFP analysis there was no main effect of Pragmatics, with activation in the Naming condition larger than in the Request condition. The sources are reported for the four peaks identified in the GFP analysis (see Figure 3)

***Peak 1 (128 ms):*** Sources of increased activation to Request compared to Naming were observed in fronto-central areas, including superior, medial, and inferior frontal gyri (BA45 and 47) bilaterally, as well as in the postcentral gyrus in the left hemisphere. The difference was also observed in temporo-parietal areas, including the inferior temporal gyrus, superior, and inferior parietal lobule bilaterally, as well as the TPJ in the right hemisphere.
***Peak 2 (180 ms):*** The differences were largest in the frontal (superior, medial, and inferior frontal gyri) and temporal areas (BA21) bilaterally, as well as in the parietal (both inferior and superior) areas in the right hemisphere.
***Peak 3 (310 ms):*** Bilateral sources were identified in the frontal (superior, medial, and inferior gyri) and inferior parietal areas bilaterally, and in the post-central and temporal areas in the right hemisphere.
***Peak 4 (468 ms):*** The last peak revealed sources in the frontal areas, especially the inferior frontal gyrus, as well as precentral and postcentral areas in the left hemisphere and superior temporal areas around the TPJ in the right hemisphere.

For the semantic condition, the GFP and the signal space analyses showed Semantics main effects and interactions (Hand > Non-Hand) only in the first two time windows (120 and 180 ms); therefore, sources were reconstructed on the difference wave (Non-Hand condition subtracted from Hand condition) only for these time windows.

***Peak 128 ms:*** Sources in the fronto-central areas including left inferior frontal gyrus and right temporal pole were identified. The superior frontal and left precentral gyrus (BA4) were activated.
***Peak 180 ms:*** Robust bilateral sources in the precentral gyrus were revealed, together with the activation in the bilateral inferior and middle frontal areas and mostly left-temporal sources.

## Discussion

Event-related brain potentials demonstrated an early and robust neurophysiological dissociation between speech acts performed with identical single words in closely matched physical settings. When words were used to Request an object, activation was near-instantaneously (latency 110–130 ms) enhanced in inferior-frontal language areas and in bilateral fronto-central sensorimotor systems, compared with a condition where the same words were used to Name objects.

Request-elicited activation was also enhanced in the right TPJ. Pragmatic effects occurred at approximately the same time as those related to semantic differences between critical word stimuli, with a range of statistical interactions between these two types of variables throughout the response epoch.

### Time course of speech act and lexico-semantic processing

Both statistical analysis of GFP and signal space topographical analysis of ERPs consistently revealed significant effects of pragmatics early on. Although these different analysis techniques led to somewhat divergent results, they are open to a coherent interpretation. A significant main effect of Pragmatics emerged in the first time window analysed (110–130 ms) and was accompanied by a near-significant (*p* = 0.054) main effect of Semantics, which became fully significant in the second (175–185 ms) time window, thus suggesting near-simultaneous pragmatic and semantic processing at these very early latencies. That both psycholinguistic factors, Pragmatics, and Semantics, were neurophysiologically reflected at early latencies was further confirmed by signal space analysis, but, in this case, by significant interaction effects of Semantics and Pragmatics in both early time windows now also involving topographical variables. This latter result provides evidence in favor of not just simultaneous but also *interactive* processing of semantic and pragmatic information. In particular, the interaction effects speak against a serial time line of semantic and pragmatic processes. Together, the results of GFP analysis and topographical analysis of ERPs indicate a predominance of pragmatic effects early on and of semantic effects slightly later, but demonstrate that, on a finer analysis level, both factors are manifested neurophysiologically together at both of the early time windows analysed.

At 200 ms after the critical word stimulus (speech act onset) and later, no significant main effects were observed in the GFP analysis for either Semantics or Pragmatics factors, including no significant modulation of N400 and P600 components. In the signal space analysis involving topographical factors, both factors, Semantics and Pragmatics, interacted with each other in the first two time windows, leaving, however, a significant Pragmatics-by-Laterality interaction in the 3rd time window (up to 335 ms) and only a marginally significant interaction of Pragmatics with the Anteriority factor in the 4th time window, thus suggesting that by 200 ms all relevant semantic information was processed, and that after 350 ms the pragmatic effects were no longer fully significant. In the context of previous findings suggesting the N400 as the main neurophysiological marker in both semantic (see Lau et al., 2008) and pragmatic domains (see Van Berkum, 2009 for a review), as well as a P600 signaling both syntactic and pragmatic effects (see Van Berkum et al., 1999; Burkhardt, 2007; Coulson and Lovett, 2010) these findings are somewhat unexpected. Note that, in previous studies, late (>350 ms latency) results were elicited by a variety of different extralinguistic experimental manipulations, ranging from matching the speaker voice with the message (Van Berkum et al., 2008) and responding to morally objectionable statements (Van Berkum et al., 2009) to discourse and world knowledge integration (Hagoort et al., 2004). It may, therefore, be that in some of the earlier studies the brain responses were dominated by rather late, task-induced “second thought”-type of processes. The present study is the first to examine the dynamics of basic speech act comprehension, emphasizing everyday conventional interaction, without relying on unexpected stimuli and comparing different pragmatic conditions to each other rather than to violation conditions. It may be that these features are critical for obtaining the early neurophysiological manifestations of pragmatic processes documented here. There are several ways in which the current experiment is different from previous research.

Firstly, most pragmatic N400 effects reported in the literature have been elicited in violation conditions, when unexpected words were used in a wider sentential, discourse, or world knowledge context. As it is not clear how far violation paradigms reflect normal language processing, in the current experiment all of the speech acts appeared in an expected context with congruent actions preceding and following the utterance (hence the lower integration processing costs). Moreover, mutual confounding of semantic and pragmatic factors was avoided by including Semantics as a separate orthogonal factor in the experimental design with well-matched stimulus categories and conditions. Previous findings show that when all the psycholinguistic and semantic properties of the stimuli are controlled, brain signatures of semantic understanding and contextual integration are present before 200 ms (Pulvermüller, 2001; Sereno et al., 2003; Barber and Kutas, 2007; Penolazzi et al., 2007; Wirth et al., 2008; Hauk et al., 2009; Pulvermüller et al., 2009). Besides, some of the effects labeled as N400 for the wider discourse integration start early at 150 ms (Van Berkum et al., 2003) so that it needs to be investigated whether such early effects are best described as parts of the N400 or as separate early responses. The early onset is in line with the early effects reported here, although the early effects seen here were clearly different from N400-like responses.

Secondly, all previous studies used syntactically complex stimuli; therefore, the heavy processing load imposed by the need for analysing and possibly re-analysing complex syntax may have delayed pragmatic effects artificially. Pragmatic P600 effects are often reported in this case, for example in experiments on integration of bridging inferences in sentences, or in a comparison of indirect requests and literal statements, using 7-word utterances (e.g., Burkhardt, 2006, 2007; Coulson and Lovett, 2010). In the current experiment single words were used leaving only contextual/pragmatic differences to explain the divergence of the ERP waves and avoiding any potential delay necessitated by processing of different types of information. Single word utterances are a standard way of communication. They are simple, and yet fully natural. Note that the choice of single word utterances (or “holophrases”) as stimuli is well-motivated and firmly rooted in the pragmatic literature (e.g., Wittgenstein, 1953; Dore, 1975; Barrett, 1982).

In the current experiment, 10 critical words were introduced by one context sentence, which could have influenced the speed of processing, by increasing statistical probability of the speech act type within a trial, thus making it more predictable. Such presentation, however, is common in real life communication. For example, in a restaurant it is natural to order several items following the waiter's question “What can I get you?” In such everyday language contexts, it would be rather unnatural to request only one item at a time expecting a new question for the next one. Therefore, the temporal dynamics revealed with this mode of stimulus presentation is relevant for everyday social communicative interaction. That said, we acknowledge that the exact timing of speech act processing in unpredictable contexts with controlled low probabilities within trial sequences may well be different and should thus be addressed in future experiments.

Importantly for the current results, even though the context was already set before the critical words appeared, no differences between Naming and Request were present prior to the critical words. The analysis of the brain activation during the presentation of simple stimuli (fixation crosses), which were presented after the sentences but before the critical words, did not reveal any significant differences between the pragmatic conditions. Therefore, it was only the presentation of word stimuli, with which typical Naming or Request actions can be performed that triggered significant pragmatic processing differences. This is not surprising given the design of the experiment, which makes the speech act of interest likely, but not certain. Although the context sentence appeared in the beginning of the trial, this context did not ultimately determine the speech act type of the utterance, for which the subsequent word was used. By using the context sentences, the speech acts of Offering assistance (e.g., “What can I get you?”) and Asking for a label (e.g., “What are these called?”) were performed. The action sequence structure for the Offer allows for different moves, including Requests for an object (such were most of the stimuli in our experiment), Rejection of the offer (e.g., by uttering “Nothing”), or Clarification (e.g., by saying “What did you say?”). These other (i.e., neither Naming nor Request) response types were implemented in least 20% of the trials. In other words, in the situation when the context sentence “What can I get you?” in our experiment was followed by “Nothing,” this latter word could not be interpreted with certainty to function either as a case of Naming or as a Request. Therefore, the context sentence was not sufficient to determine the Speech act type at the point when the critical word appeared, and thus, the disambiguation point was at the onset of the critical word, which was used to perform either a Naming or a Request action. Clearly, in the absence of the critical word, no Naming or Requesting could be performed.

In summary, these results support the model of near-simultaneous processing of subtypes of psycholinguistic information (Marslen-Wilson, 1975; Pulvermüller et al., 2009; Shtyrov, 2010), which predicts that all the relevant information about all the levels of language processing (phonological, lexical and morphological, semantic, syntactic, and pragmatic) is first accessed within the first 200 ms of the critical stimulus onset. All the processing is done in a parallel rather than consecutive fashion, and leads to interaction between the various processing levels, which was shown here for the levels of semantics and pragmatics. The re-computation and post-processing in the later time windows is not necessary but can happen, giving rise to task-related post-understanding (e.g., decision about semantic-conceptual correctness, voice identity, etc.) and other second thought type of processing, if a more complex stimulus is encountered. In the current experiment, both semantic and pragmatic effects were registered within the first 200 ms, exhibiting interactions between semantic and pragmatic levels in the first two time windows, with most effects remaining only marginally significant by 400–500 ms, given that simple, predictable, psycholinguistically controlled single word stimuli were used.

These results do contradict two premises of serial and cascaded psycholinguistic models. Firstly, the observed interactions between semantics and pragmatics in early time windows challenge the prediction that the processing is consecutive and supported by separate processing components. Secondly, the effects of pragmatics starting concurrently with, or even before semantics, contradict the idea of successive processing, when a higher (pragmatic) level is not reached until the lower (semantic) level information is processed.

Note that, in view of the recent discussion of possible temporal imprecision—in the range of 20–30 ms—introduced by low-pass filtering of EEG data and associated filter ringing artifacts (VanRullen, 2011; Widmann and Schröger, 2012), no filter ringing artifacts were detected in the current analysis. Importantly, the general conclusions that (1) pragmatic effects appear extremely early and (2) early semantic effects documented in previous literature could be replicated in this study, remain unaffected, even if the exact timing of the peaks may be subject to modification (±20–30 ms) in future studies.

It is also important to point out that the current result is obtained for the comprehension of speech acts by an external observer. Although it is likely that a similar pattern of activity may take place in individuals who participate in the interaction *per se* (i.e., Speaker and Partner), future studies are necessary to investigate the production and comprehension of speech acts from the perspective of the immediate agents involved.

### Brain loci of speech act processing

With respect to the neural bases of speech act comprehension, throughout all investigated time windows, starting from 110 ms, the speech act of Requesting elicited generally higher ERP amplitudes. L1 minimum norm estimation of cortical sources suggested that two specific regions of cortex—bilateral fronto-central and right temporo-parietal areas—showed enhanced activation during processing Requests compared with Naming.

Together the signal space analysis with the topographical factors and the reconstruction of sources of activation indicated the involvement of the bilateral IFG and sensorimotor areas (pre-/post-central gyri) in all the time windows. The activation in these regions is best explained by the action-related nature of a Request. Since Requests are embedded in an action sequence structure (Fritz and Hundsnurscher, 1994), which is more complex in the case of a Request than in Naming (see “Introduction” and Figure 1), the fronto-central cortex could be the neural basis supporting the knowledge about and implementation of these action-sequences. Sequence processing and computation of hierarchical action structure is likely to be carried out by Broca's area and premotor cortex (in interaction with other areas and functions; see, e.g., Pulvermüller and Fadiga, 2010) in a way similar to the storage of action-semantics.

Another observation from the source reconstruction was the engagement of areas around the right posterior superior temporal sulcus (pSTS), inferior parietal sulcus (IPS), and TPJ. These areas are often implicated in social inferencing (IPS) and the recognition of communicative traits (rTPJ) and intentions (pSTS) (Sassa et al., 2007; Van Overwalle et al., 2009; Noordzij et al., 2010). As to the activation in this region, Fogassi et al. (2005) have previously suggested that inferior parietal areas play a role in intention recognition through the involvement of mirror neurons that discriminate identical motor acts according to the action goal they achieve. This mechanism could be relevant for speech act comprehension in that speech acts are embedded in larger action sequences with different communicative goals. Similarly to the encoding of motor acts by predicting the goal of the action in the experiments of Fogassi and colleagues, speech acts in this study could be differentiated by predicting the actions they are followed by. Noordzij and colleagues have specifically attributed the recognition of the intent of the communicative actions, both in the communicator and the comprehender, to the right posterior STS (Noordzij et al., 2010). Moreover, the temporal dynamics of the activation in this temporo-parietal region in the current experiment, namely its early start, resembles that revealed by Van Overwalle and colleagues in an EEG experiment showing that trait inferencing in goal-directed behavior engages the right TPJ, starting at 150 ms (Van Overwalle et al., 2009). However, bearing in mind that the spatial resolution of the EEG source reconstruction only allows coarse localization, these activation patterns and their implications for speech act processing should be taken as indicative and investigated with spatially more precise metabolic imaging techniques (fMRI) and higher-density neurophysiological recordings (combined EEG/MEG) in future studies.

Only in one time window did the topographical analysis reveal greater activation to Naming than to Request. In the 2nd time window centered around 180 ms, pragmatic effects were revealed specifically at the anterior-peripheral sites in the “Hand-related” semantic category. The spatially specific dominance of Naming in this time window might suggest that the speech act of Naming activated the mechanism of retrieval of the semantic information about the named objects in temporal regions. However, this finding was not supported by the GFP results for the two pragmatic factors, and in the absence of confirmatory sources of activation, any conclusions about naming-specific activation remain suggestive.

Thus, comprehension of speech acts activates cortical areas that support crucial components of the communicative function they bear. In the case of Naming, the main function is to refer to an object by using a linguistic expression, which activates semantic knowledge linking the two. For this process, temporal areas, interfacing between visual and language areas seem most relevant. This study provided limited evidence for such activation. However, in the case of Request, the main function is to make the Partner perform an action, which requires the knowledge of social actions the speech act may bring about as part of its action sequence structure (Figure 1), and such pragmatic linkage to action knowledge was reflected at the level of the brain by activation in the inferior frontal and sensorimotor cortices. Additionally, given that the action of Requesting presupposes that the Speaker predicts and recognizes the goals and intentions of these social actions (e.g., differentiating between denying and refusing the request) of the Partner, the engagement of the right temporo-parietal cortex known to be relevant for interaction and social knowledge was observed. All of these activations were recorded while the participants simply observed and understood social communicative interaction.

The finding that speech act processing engages the sensorimotor system and the inferior parietal cortex, possibly reflecting pragmatically related access to action knowledge, and intentions and assumptions, respectively, resembles a previous report by Van Ackeren and colleagues (2012) who found that the same brain systems were active for processing indirect requests (critical condition) but not literal statements (control condition). Based on this fMRI study alone, it is, however, not clear whether sensorimotor and inferior-parietal activations appear at early or late latencies. Unlike the EEG technique applied here, the nature of fMRI makes it impossible to draw conclusions about the time course of cognitive processes with millisecond precision. Such fMRI results are therefore open to an interpretation of results in terms of post-understanding epiphenomenal activations. Furthermore, the comparison of indirect requests with statements also leaves it open to what degree the differential brain activations reflected speech act processing *per se* or rather the indirectness of such acts. Van Ackeren and colleagues interpret the effects in their study in terms of indirectness; however, the speech act types of the stimuli, requests vs. statements, were also different. The present results show clearly that sensorimotor and inferior-parietal activations occur early, which provides an argument that they are related to comprehension processes. Crucially, sensorimotor and inferior parietal activations here represent a signature of the speech act of Requesting, independent of directness or indirectness. This novel finding suggests that the above interpretation in terms of indirectness needs to be revised in favor of speech act types as the critical factor. However, both studies taken together now converge on the finding that during Request processing, sensorimotor and theory-of-mind (TOM) areas are engaged, which possibly reflects access to action knowledge and social-interaction information relevant in the comprehension process.

## Conclusions

Speech acts of Naming and Request performed by uttering a single word were compared in an EEG experiment. Activation differences were found as early as ~100–200 ms after the onset of the word, with which the critical speech acts were performed, when, as suggested by the significant interaction in the topographical analysis of the first time window, processing of both pragmatic and semantic aspects of meaning began. These findings are not compatible with the predictions of serial processing models but provide strong evidence for near-simultaneous access to psycholinguistic information in the mind and brain in speech act comprehension.

Further analysis of the response amplitude and topography in the signal space and the activation sources suggested that the neural organization of speech act processing involves the left perisylvian cortex (for linguistic processing), bilateral fronto-central cortex (for processing action sequence structures), and right temporo-parietal cortex (for processing further interaction knowledge and aspects of theory of mind). These brain areas appear to particularly contribute to specific types of speech acts; namely Requests, were found to activate sensorimotor cortex and right temporo-parietal regions more strongly than Naming.

### Conflict of interest statement

The authors declare that the research was conducted in the absence of any commercial or financial relationships that could be construed as a potential conflict of interest.
